# Did Climate Change Influence the Emergence, Transmission, and Expression of the COVID-19 Pandemic?

**DOI:** 10.3389/fmed.2021.769208

**Published:** 2021-12-08

**Authors:** Saloni Gupta, Barry T. Rouse, Pranita P. Sarangi

**Affiliations:** ^1^Department of Biosciences and Bioengineering, Indian Institute of Technology Roorkee, Roorkee, India; ^2^Department of Biomedical and Diagnostic Sciences, College of Veterinary Medicine, University of Tennessee, Knoxville, Knoxville, TN, United States

**Keywords:** COVID-19, climate change, host immunity, reservoirs, vectors

## Abstract

The human race has survived many epidemics and pandemics that have emerged and reemerged throughout history. The novel coronavirus Severe Acute Respiratory Syndrome SARS-CoV-2/COVID-19 is the latest pandemic and this has caused major health and socioeconomic problems in almost all communities of the world. The origin of the virus is still in dispute but most likely, the virus emerged from the bats and also may involve an intermediate host before affecting humans. Several other factors also may have affected the emergence and outcome of the infection but in this review, we make a case for a possible role of climate change. The rise in industrialization-related human activities has created a marked imbalance in the homeostasis of environmental factors such as temperature and other weather and these might even have imposed conditions for the emergence of future coronavirus cycles. An attempt is made in this review to explore the effect of ongoing climate changes and discuss if these changes had a role in facilitating the emergence, transmission, and even the expression of the COVID-19 pandemic. We surmise that pandemics will be more frequent in the future and more severely impactful unless climate changes are mitigated.

## Introduction

**Figure 1:**
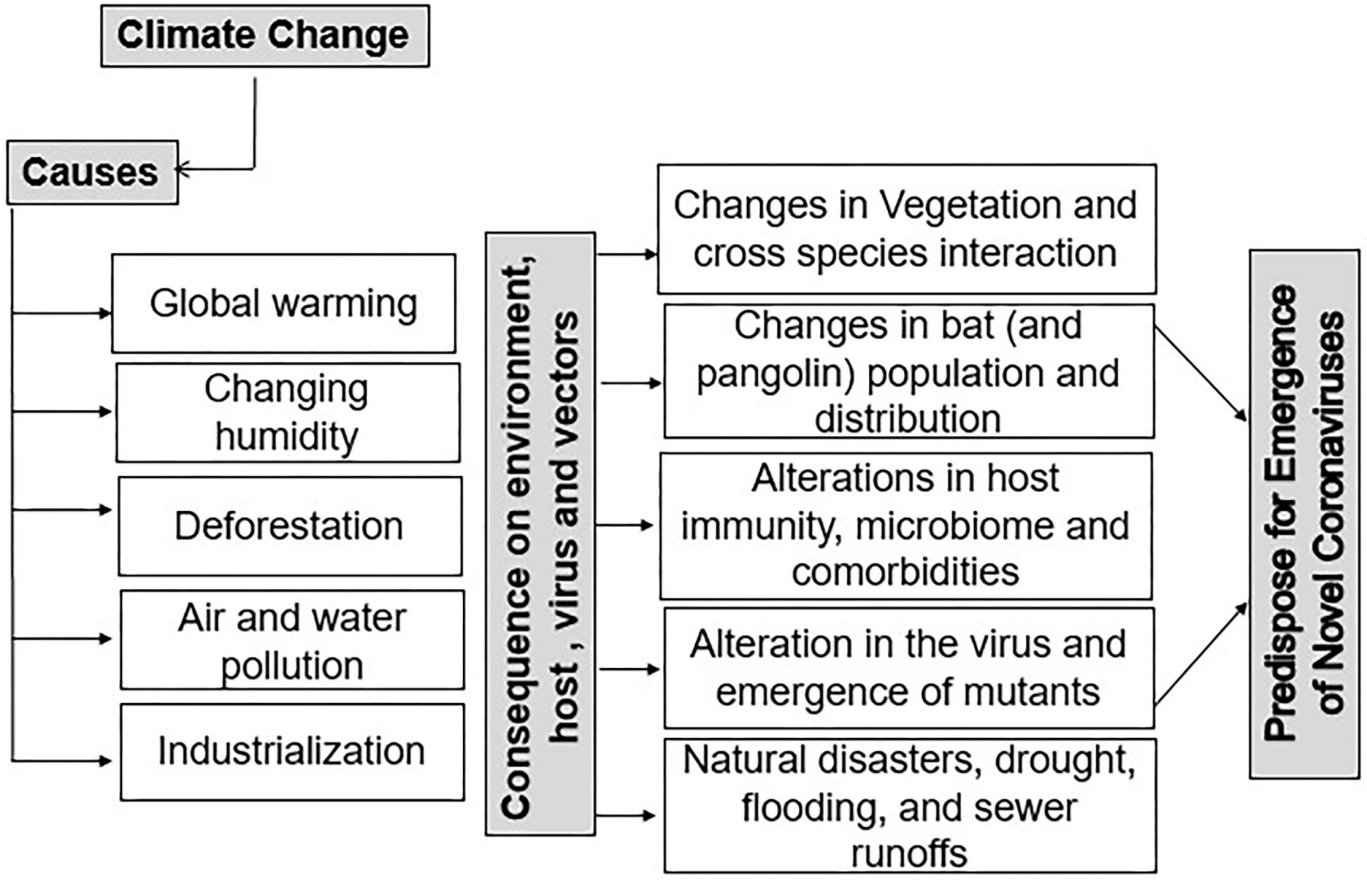
Climatic change as a driver for the emergence of novel coronaviruses. Changes in different climatic parameters such as temperature and humidity are shown to impact the emergence of novel coronaviruses by altering the host, vectors, and the virus itself. Changing climate has significantly affected the habitat distribution of vectors and carriers (i.e., bat and pangolin populations). Changes in construction, deforestation, frequent natural calamities, and an increase in air pollution have also affected host immunity, virus transmission efficiency, and cross-species interactions and have contributed to the emergence of novel coronaviruses.

**Figure 2:**
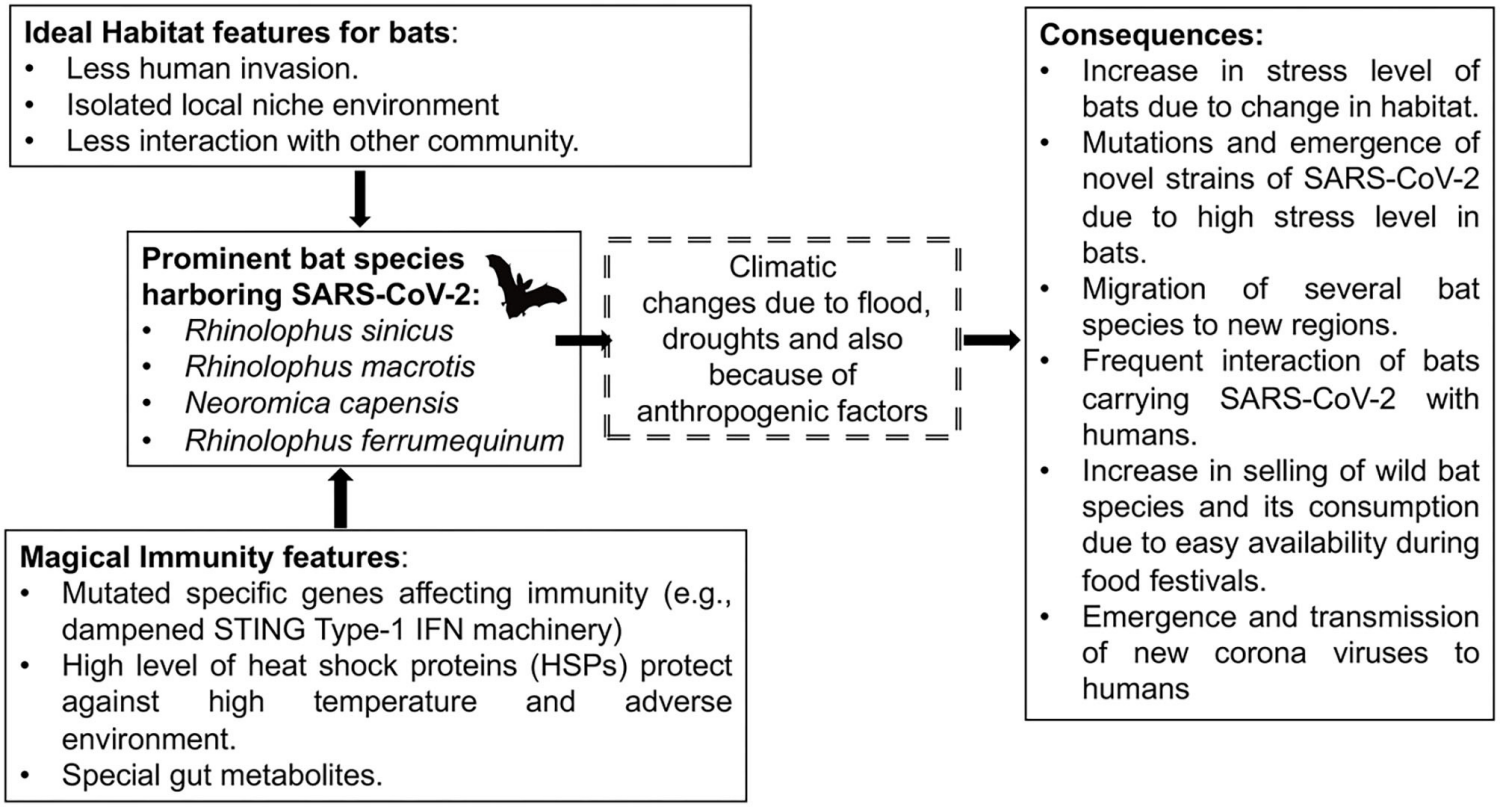
Effect of climate change on bats and its habitat and its correlation with SARS-CoV-2 emergence. Many natural and anthropogenic interventions in the native habitat of bats had increased the probability of their contact with humans making mankind more susceptible to SARS-CoV-2. Climatic variations and anthropogenic factors have resulted in increasing stress responses in bats that carry various coronaviruses. Such changes in bat physiology have helped in creating novel mutants capable of infecting humans.

Statistical analysis by the John Hopkins School of Medicine of the ongoing COVID-19 pandemic on different nations worldwide has quantified the evidence that the novel Severe Acute Respiratory Syndrome Coronavirus-2 (SARS-CoV-2) has devastated the global economy and health care systems in almost all countries (1, 2). It has affected at least 220 countries and territories, with more than 245,548,901 people infected and more than 4,983,723 deaths, as of 27 October 2021 (2). A recent statistical analysis on the US population showed that COVID-19 ranks as the third major cause of mortality for children and adults (697.5 deaths/million) after heart disorders (1,287.7 deaths/million) and cancer (1,219.8 deaths/million) (3, 4). Hopefully, we can be optimistic about the eventual control of COVID-19 since multiple effective vaccines have been produced and these have considerably reduced the outcome and impact of the pandemic in countries where they have been available and widely used. Although the durability of vaccine-induced immunity and its protective efficacy against emerging mutants is yet to be fully understood, there are excellent reasons to anticipate worldwide control of the pandemic once vaccination becomes readily available and assuming that all communities embrace their use (5). Conceivably, the global catastrophe caused by COVID-19 may become a past bad memory. However, given the circumstances of our world with its changing climate and its anthropocentric stresses such as deforestation, industrialization, and pollution we can anticipate that new pandemics of coronaviruses and other infections will remain on the menu (Figure 1) (6–8). Supporting this hypothesis, many lines of evidence indicate that climatic and weather changes due to natural (e.g., forest fires, volcanoes, and solar radiations, increased cyclones and floods) and modern civilization associated reasons as described above have impacted human health, changing susceptibility to infections, the distribution of reservoirs, carriers, and their exposure to a wider range of host population (7). With respect to the host, reports showed that the current changes in environmental factors have increased the incidences of lifestyle diseases such as diabetes in humans and have affected several aspects of host immunity to the SARS-CoV-2 virus, such as expression levels of the viral ACE-2 receptor (9–11). Importantly, changes in environmental temperatures combined with changes in human activities have significantly impacted the migration of the bat species that carry coronaviruses in specific geographical regions, setting the stage for the emergence of novel viruses and their transmission to the human host (Figure 2) (6). Similarly, the transmission of the SARS-CoV-2 via air, water, and infectious aerosols also may influenced by global climate changes (12–14). In this review, we examine existing evidence as to how climate change and human activities may have facilitated the COVID-19 pandemic by influencing the above-mentioned factors such as reservoirs of the virus, its transmission, and the susceptibility of the human host above factors. Better awareness and efforts to reduce the progression of such changes will help in minimizing the impact of the next pandemic when it inevitably emerges.

## The Climate Is Changing

In the current era of technological advancements and industrialization, the consensus in the scientific community is that the climate is changing and is affecting population growth, land use, and, as we speculate in this article, the emergence and success of new pathogens, such as the current COVID-19 pandemic (8). It can readily be demonstrated that recent events, including the melting of ice caps, increased forest fires, and frequent new temperature records, show that the planet is gradually warming (15). In fact, an Intergovernmental Panel on Climate Change (IPCC) predicted that the average global temperature would rise by 1.5 to 4°C in the coming decades (16, 17). Moreover, the 2014 National Climate Assessment report documented that historically wet areas are becoming wetter while historically dry areas are getting arider, overall, due to global warming (18, 19). These rising temperatures, irregular precipitation, increased atmospheric CO_2_ levels, and cloud cover have all played a role in causing changes in vegetation and plant growth patterns, and this has affected the distribution of vector organisms, such as those that can transmit pathogens such as SARS-CoV-2 (Figure 1) (6, 8, 20–23). One of the causes, which have set in motion the chain reaction leading to climate change, seems to be changing human activities. Activities such as farming, deforestation, and infrastructure growth have transformed the native habitats of organisms, particularly in tropical areas, due to their high biodiversity of flora and fauna (20, 24). These human activities have increased the opportunity for cross-species infections and the onset of new pandemics such as COVID-19 (20, 24–27). Also of note, it is evident that increased use of personal protective equipment made up of plastics and polymers being used to control the COVID-19 pandemic could have a negative impact on the climate. Thus, if not used judicially, or disposed of in an environment-friendly manner, these plastics and polymers may be detrimental and could perhaps facilitate the emergence of novel infections (28). Highlighting this concern, many reports have advocated for the preservation of intact ecological niches and their biodiversity to decrease the risk of new emerging infections (29–34). In this brief review, we provide evidence and, when possible, mechanistic explanations of how climate change could have contributed to the emergence, transmission, and perhaps even the disease consequences of the COVID-19 pandemic.

## Is Climate Change Driving the Emergence of Novel Coronaviruses?

Although the origin of the pandemic coronavirus is in debate, bats are a likely point source. Historically and until the SARS epidemic in 2003, which originated in late 2002 in China, bats were not known to carry coronaviruses (35–38). However, since that time, several species of bats have been shown to harbor multiple coronaviruses including, SARS-CoV-1 in 2003, MERS in 2012, and SARS-CoV-2 in 2019 (38–41). Bats are common across the world and represent 1,423 of the more than 6,400 known mammalian species (42). Over some 64 million years of adaptive evolution, bats have acquired unique immune defenses that allow them untroubled infection with an abundance of many different virus infections (42). Unfortunately, some of these are zoonotic and can cause illness and death when they infect humans. These include the three coronaviruses already mentioned and likely some of the common cold-causing coronaviruses as well as Nipah and Ebola viruses (42).

The evolutionary changes that permit bats to accommodate so many viral infections include mutations in specific genes that affect their innate and adaptive immune systems. For example, the immune tolerance of bats to high exposure levels of RNA viruses was shown to be influenced by dampened STING-dependent type-I interferon gene expression (43). Secondly, bats display enhanced autophagy and an efficient ABCB1 efflux mechanism for blocking DNA damaging substances, and reduced reactive oxygen species (ROS) production that controls unwanted inflammation (44–46). Bats also express high levels of heat shock proteins, protecting them against high temperature and oxidative stress by chaperoning viral proteins and providing them the potential to induce mutations in viruses (47). Several reports show that bats have dampened the functions of several sensors (e.g., NLRP3 and PYHIN gene family members such as AIM2 and IFI16) at both the transcription and protein levels and these sense the danger signals induced by potential pathogens and assist in fighting stress and infection via endogenous anti-microbial mechanisms (48). Another intriguing anti-microbial activity is mediated by their gut microbiota, which releases metabolites that help shape their unique immune defenses (49). Thus, the unique immune system in bats not only enables them to serve as asymptomatic carriers but also provides a suitable reservoir for the coronaviruses to undergo novel mutations that could make them more dangerous when transmitted to other species, including mankind. The evidence that bats act as viral reservoirs is irrefutable, but the question that needs consideration is how climate change may have made bats more relevant as a source of novel pandemics such as the one of current concern.

Evidence suggests that the changing climate is influencing the transformation of native habitats from shrublands to savannah as occurred in southern Yunnan, the likely origin of COVID-19 (Figure 2) (6). These plant habitat transformations caused changes in the species distribution and population densities of bats and favored the expansion of those bat species that carry multiple coronaviruses (6). For example, more than 40 strains of forest bats migrated to the woodlands and shrublands of the Yunnan region over many years and these have nurtured over 100 additional species of bat-borne viruses (50). Conceivably, increased human contact with such bats also may have occurred because of changing human recreational pursuits or cultural activities such as dietary adventures in food markets where exotic products are sold (6, 51). Along with changes in bat populations, some researchers have suggested that ongoing climatic changes might have resulted in the migration of pangolins, a likely intermediate host of SARS-CoV-2 and conceivably, coronaviruses could evolve in pangolins to become more pathogenic for humans (6, 50, 52–55). In addition to a change in habitats, climate changes such as floods and droughts have caused humans and animals to change habits and migration patterns leading to closer contact with sources of new infections (56). This may have happened in the Wuhan area, which has had some extreme droughts in recent decades (56). Of note, climatic alterations such as frequent flooding, heatwaves, pollutants, and urbanization have severely decreased the wetland regions in Wuhan, China, restricting food production and the availability of land, thereby forcing humans to find new food sources and share space with new potential sources of infections (57–60). In support of this concept, many patients admitted to hospitals with SARS-CoV-2 infection were found to have a connection with the wet animal and seafood wholesale market in Wuhan, China (61). That climate change helped set the stage for the emergence of SARS-CoV-2 from bats remains to be proven, but circumstantial evidence continues to accumulate. Climate change also might act to make the transmission of the novel SARS-CoV-2 C more likely.

## Effect of Climate-Changing Factors on the Transmissibility of SARS-CoV-2

SARS-CoV-2 is most commonly transmitted via infectious droplets expelled from infected persons resulting in infection of the respiratory tract of nearby susceptible persons (62). Climate change might have contributed to facilitating this transmission in several ways. Some climate conditions such as increased rainfall and violent weather resulting in floods and home loss have caused people to assemble closer together, facilitating the opportunity for infection with high doses of virus and a situation that results in more severe infections (63). This is evident by the concentration of SARS-CoV-2 in population-dense areas. By way of contrast, climate change has, in some places, led to desertification and this too could have contributed to the creation of crowded communities inhospitable areas making them more prone to infections (19, 64, 65). In addition, these peoples also may experience dust-mediated damage to the respiratory tract and increase access of virus to the lower respiratory tract with consequent more severe disease (66–70).

One of the more consequential results of climate change that affects transmission efficacy may be the increase in temperature and relative humidity, creating conditions that favor the long-term survival of infectious particles in the environment (71, 72). Temperature and humidity have been shown to affect the duration of particulate matter suspended in the air and influence virus transmission (73). For example, an increase in humidity elevates the rate of toxic chemical agents in the air and along with this, provides a suitable environment for more viral organisms such as novel coronaviruses to propagate and cause respiratory diseases (74). In experimental animal studies, higher temperatures were shown to alter viral replication and transmission under both *in vitro* and *in vivo* conditions, but whether the small temperature increases associated with climate change are relevant to this scenario needs further investigation (75). Other than temperature and humidity, there has been a significant increase in anthropogenic activities that have elevated mineral dust, combustion processes, biomass burning, and plant/microbial materials, all of which have increased the frequency of aerosols and particulate matters in the atmosphere (76). The aerosols and particulate matter are known to affect the global climate by altering the radiative properties of the atmosphere and they could also act as effective SARS-CoV-2 carriers (12, 77–82). Overall, many climatic factors could have contributed to facilitating viral transmission in the COVID-19 pandemic, but further studies are warranted to document the mechanisms by which the effects are mediated.

## Could Climate Change Be Contributing to Making Humans More Susceptible to COVID-19 Infection?

An unanswered question is if climate change has caused humans to be more susceptible to any viral infections, but especially SARS-CoV-2. As we and others have discussed in reviews, whether or not any virus infection results in a clinically relevant response in the infected host depends on multiple factors that affect the interaction between the virus and the host as well as environmental factors during infection (83). Climate change has clearly affected the environment, as well as the distribution of carriers and vectors, but the challenge is to explain if and how such changes impact mechanistically on the agent-host environment interaction. One highly relevant variable that affects the outcome of any infection is the virus exposure dose (84). The dose of exposure, as mentioned in the above section, is both relevant to transmission as well as the outcome of infection as it relates to SARS-CoV-2 (84). Certain climate change effects could act to raise the dose and formulation of infection with the more severe consequences occurring when infected with high doses of the correct particle size in aerosols (85). According to the WHO, the primary mode of SARS-CoV-2 transmission is via infectious aerosols such as droplet infections, which might be playing an important role in elevating coronavirus disease severity (86). What also is possible, but not yet firmly established, is if climate change is acting to impair the efficacy of the host's defenses against virus infection.

A major host factor that affects whether or not a virus infection results in disease or is unobserved is the efficacy of host innate and adaptive immune responses. As mentioned in the above section, a concentrated population due to floods and droughts also may experience food deprivation and other infectious diseases which affect their immunity and may act to blunt the efficiency of immune system responses, making it difficult to control even modest doses of SARS-CoV-2 infection (87). The WHO has reported that insufficient nutrition leads to impaired immunity and malfunction (88). For example, some studies have shown a correlation between Vitamin D deficiency and an increased risk of viral infections (89). In addition, air pollutants and greenhouse gases may be disabling antiviral immune systems, and this mechanism was suspected to explain why the initial COVID-19 outbreak in Italy occurred in Lombardy - an industrial area whose population is known to have a problem with vitamin D deficiency, especially in their cloudy winters (68, 90, 91). This could be associated with high levels of greenhouse gases and anthropogenic aerosols, which cause UV-B absorption and scattering by ozone and thus less availability for cutaneous synthesis of vitamin D, which could lead to its deficiency (92–95).

Many have connected climate changes with an effect on the composition of the gut microbiome and this change may further explain impaired immune defenses against systemic virus infections (15, 96, 97). The human gut microbiome consists of about 1,000 species of bacteria and 100 trillion organisms, which are involved in vitamin production, metabolizing food, and the regulation of immune responses against pathogens (96, 98, 99). For example, *in vivo* studies using mice models have shown that the generation of immune reactions dominated by Th17 CD4+ pro-inflammatory T cells, which are involved in resistance to some infections, is influenced by the presence of Bacteroides species in their gut (100). In other studies, the nature of the diet, particularly its fiber and fat composition, was shown to affect the ability of the host to generate regulatory T cell responses, an effect which would serve to dampen immune pathological responses to virus infections, such as occur in severe responses to SARS-CoV-2 (101, 102). Climate change is expected, via effects on nutrition, soil microbiota, and living conditions, to have changed the nature of the microbiome in many communities. In addition, recent studies provide evidence that climate change-driven weather conditions and agricultural practices such as increased antibiotic usage in food production have affected the plant biomass and modified dietary plans likely affecting the composition of the microbiota (103–105). Similarly, climatic factors are also changing the soil microbiota, which could also alter food quality and indirectly influence could the gut microbiome (103, 104, 106–108).

The challenge is to explain how the proposed microbiome differences induced by climate change could affect host immunity. One reason could be an effect on the expression levels and distribution of the ACE2 molecules that are the receptors used by SARS-CoV-2 to infect cells (109–111). One report indicated that the distribution of ACE2 receptors could be affected by physiological temperature by way of differential stimulation by heat shock proteins (HSP72), that are upregulated in human cells when the temperature exceeds the normal range (9, 112). Another factor by which climate change could influence the efficacy of immunity to SARS-CoV-2 is exposure to other comorbidities (7). A prime candidate to consider is diabetes, a well-known susceptibility factor for severe COVID-19 infection (11, 113). Some evidence has linked ambient temperature with an increased rate of diabetic cases (10, 114, 115). For example, a study by Blauw et al. shows that an increase of 1C may account for more than 100,000 diabetic individuals/year in the United States due to dehydration and thermal stress associated with alteration in insulin absorption and diffusion (116). It is also known that comorbid conditions modulate the immunological functions of the host and alter their ability to defend against viral diseases (11). These diabetic individuals may have many immune problems that include neutrophil chemotaxis, phagocytosis, microbicidal activity, blunted anti-viral responses along with impaired T-cell immunity, and hyper-inflammatory response. These all make diabetics potentially more susceptible to infections, including SARS-CoV-2 (117, 118).

With the growing population, reports suggest that higher population densities along with environmental factors such as CO_2_ emission may facilitate the transmission of the novel viruses (119). Supporting this concept, a recent finding showed that the mortality and rate of transmission of SARS-CoV-2 virus were higher in densely populated areas of India compared to areas with lower population densities (120). However, there were also other factors that came into play such as health status, the average age of people residing the area as well as different economic conditions. A separate study published from the Johns Hopkins Bloomberg School of Public Health, with data obtained from 913 urban counties of the USA, suggested that the death rate had an inverse correlation with population density except for metropolitan regions where a higher rate of infection and mortality was observed (121).

Accumulating evidence has indicated that the changing climate could have made some persons more susceptible to infection during the COVID-19 pandemic because the efficacy of their immune defenses has been compromised by several potential mechanisms. The most relevant of these could be changing nutrition because of food shortages or dietary changes imposed by changes in food access and lifestyle habits. Further investigation on how climate change, comorbidities, and nutrition alter various aspects of host defenses and viral pathogenesis will help clinicians and researchers to demystify and predict our susceptibility to future pandemics. The emergence and severity of viral epidemics and pandemics is dependent on the development of strong herd immunity. To date, few studies have shown the impact of changing climate on vaccine-preventable diseases (122). However, with respect to SARS-CoV-2, the effect of changing climatic factors on the development of herd immunity to COVID-19 still questionable and require further investigation (123–125).

## Are the Changes in the Air and Water Quality Contributing to the Transmissibility and Pathogenesis of SARS-CoV-2 Infections?

Hopefully, we can accept the evidence that climate changes have contributed to the emergence, transmission, and perhaps even the severity of the COVID-19 pandemic, but additional ongoing problems are occurring in the modern world that also are impacting the pattern of the pandemic. One of these is environmental pollution. Studies to measure the impact of pollution on the SARS-CoV-2 infection and transmission are accumulating. We have already mentioned the situation in Italy where the SARS-CoV-2 outbreak might have been linked to an environmental pollution effect (68–70). In addition, studies in China could associate regions with higher levels of other air pollutants such as SO_2_, CO_2_, PM_10_, and O_3_ in the atmosphere with a higher risk of respiratory disorders, including SARS-CoV-2 (66, 67). PM_10_ is the size (10 microns or below) of particulate matter which can be inhaled and penetrates deep into the lungs resulting in the most consequential SARS-CoV-2 viral lesions (13, 126). These PM_10_ particles have increased in the atmosphere because of fossil fuel use and other human activities. (126). Several reports from China have shown a positive correlation for PM_2.5_ materials in the air with SARS-CoV-2 cases associated with a higher increase in air-borne PM_2.5_ compared to PM_103_ (66). In addition to PM_2.5_, recent evidence from multiple countries has associated NO_2_ levels with COVID-19 fatalities. (67, 127, 128). Many studies have shown that climate change could affect the abundance of many environmental pollutants such as aerosols as well, and these were recently shown to influence SARS-CoV-2 transmission and disease severity (12, 77–80, 129–133). For example, virus aerosols can be formed by the fusion of SARS-CoV-2 with dry solid or liquid particles that are generated by an infected person's respiratory secretions, exhaled gas, and feces (134). Although they could be unstable, still they could cause extensive infection via the conjunctiva, damaged skin, or the digestive system (134, 135).

Another potential susceptibility factor for SARS-CoV-2 infection is water pollution, especially in areas where climate change has affected the quality of the water supply. Water can be a source of COVID-19 RNA, which has been detected in sewage (14, 63, 136). Although RNA is usually considered to be fragile, recent studies showed that sewer water from multiple sites in Paris indicated that infectious viral RNA could remain in sewer water for up to seven days without degradation (136, 137). This resistance could be attributed to a strong interaction between nucleoproteins and viral RNA (136, 137). Such observations were also supported by studies from other parts of the globe, such as the Netherlands and Australia (70, 137–139). In addition, SARS-CoV, the cousin virus of SARS-CoV-2, was found to be transmitted from aerosolized wastewater when the infection outbreak occurred in Hong Kong (140, 141). However, it is yet to be proven that infectious SARS-CoV-2 has been transmitted in contaminated water.

## Conclusion

The occurrence and impact of the still ongoing COVID-19 pandemic have shocked the world. Perhaps it has been even more shocking to those who do not believe that our world is undergoing climate change and that mankind plays a large role in these changes. However, we have made the case that climate change may have contributed to the emergence and transmission and likely even to some of the clinical consequences of SARS-CoV-2 infection. The reasons include evidence that the likely reservoir source of coronaviruses for human infection has increased in number because of climate-induced changes in vegetation, and human activities bringing them into closer contact with bats and animals such as pangolins that could represent the intermediate hosts. This interaction in the case of COVID-19 might have happened in the field or markets, although there are still unproven suggestions that the initial viral pathogen may have escaped from a laboratory (142). Whatever the initial emergence source, we also have made the case that climate change is acting to facilitate transmission between infected and uninfected persons. The case for this largely comes from weather changes causing certain groups to live in more concentrated situations, the temperature and humidity changes to favor viral survival, and the effects of industrial pollution to cause persons to cough and sneeze and create highly infectious aerosols. We contend that climate change is helping set the stage for more severe manifestations of infection. The case for this situation is harder to make and is mostly indirect. However, climate change has caused some communities to change their nutrition both in the amount consumed and the quality of their diet. The effects become manifest oftentimes by changes imposed on the gut microbiome that in turn influence the type of immune responses made. Other effects act by way of the change that climate change has had on comorbidities such as the frequency of diabetes and the prevalence of mammalian vectors, such as bats that transmit other infections that change susceptibility to SARS-CoV-2.

In conclusion, all of the above-mentioned information should be considered a wake-up message for decreasing deforestation, worldwide greenhouse emissions, and controlling the transmission of pathogens. Also, to reduce the risk of upcoming zoonotic spillovers, it is very important to spread knowledge about measures to protect natural niches and habitats, to create and impose strong rules and regulations on persons involved in wildlife hunting and trade. This includes setting animal welfare standards on farms, markets, and transport vehicles and preventing high-zoonotic-risk food and medicinal customs. Additionally, to reduce the risk of future pandemics, there is an urgent need for global measures such as better health policies, touch-less patient care systems, reduced deforestation and maintaining the natural environment. Similarly, further data collection should be facilitated to understand the correlation between environmental dynamics and the emergence of novel coronaviruses.

## Author Contributions

SG surveyed the literature and wrote the manuscript. PS edited and organized the manuscript. BR helped in logically presenting the ideas, wrote, and edited the manuscript. All authors contributed to the article and approved the submitted version.

## Funding

This work was funded by FIG-100643 to PS, CSIR, Govt. of India to SG, and R01 EY005093 to BR.

## Conflict of Interest

The authors declare that the research was conducted in the absence of any commercial or financial relationships that could be construed as a potential conflict of interest.

## Publisher's Note

All claims expressed in this article are solely those of the authors and do not necessarily represent those of their affiliated organizations, or those of the publisher, the editors and the reviewers. Any product that may be evaluated in this article, or claim that may be made by its manufacturer, is not guaranteed or endorsed by the publisher.
